# Predictors of health-related quality of life after coronary artery bypass graft surgery

**DOI:** 10.1038/s41598-022-20414-1

**Published:** 2022-09-27

**Authors:** Hwasoon Kim, Sun Hyoung Bae, Sang-Hyun Lim, Jin-Hee Park

**Affiliations:** 1 College of Nursing, Ajou University, 164 World cup-ro, Yeongtong-gu, Suwon, Republic of Korea; 2 College of Nursing, Research Institute of Nursing Science, Ajou University, 164 World cup-ro, Yeongtong-gu, Suwon, Republic of Korea; 3 Department of Thoracic & Cardiovascular Surgery, Ajou University School of Medicine, 164 World cup-ro, Yeongtong-gu, Suwon, Republic of Korea

**Keywords:** Quality of life, Cardiology, Health care

## Abstract

Health-related quality of life (HRQOL) is a multifactorial concept in assessing physical and mental health. This study was performed to evaluate the HRQOL of patients undergoing coronary artery bypass graft (CABG) surgery and the predictors of HRQOL in patients until 1 year after surgery. This cross-sectional study included 110 consecutive patients who underwent elective CABG in a medical center in South Korea. The Short-Form Health Survey, cardiac symptom survey, cardiac self-efficacy, and the Interpersonal Support Evaluation List-12 were used to measure the HRQOL, symptom experience, self-efficacy, and social support, respectively. The regression model explained 42% of the variance in the participants' physical HRQOL. The predictors of the physical HRQOL include the presence of a spouse, post-CABG duration, symptom experience, and self-efficacy. The regression model explained 36% of the variance in the participants' mental HRQOL. The predictors of the mental HRQOL included perceived health status, self-efficacy, and social support. The predictive factors for HRQOL after CABG were the presence of a spouse, post-CABG duration, symptom experience, self-efficacy, and social support. Furthermore, a suitable program and nursing interventions could be implemented to improve the HRQOL of post-CABG patients.

## Introduction

Coronary artery bypass grafting (CABG), a primary treatment for patients with coronary artery disease (CAD), such as angina pectoris and myocardial infarction, is performed to improve cardiac perfusion by bypassing the blocked arteries and creating new pathways, using autologous blood vessels, for blood flow^1^. Despite some disadvantages, such as the need for intraoperative median sternotomy and cardiopulmonary bypass, CABG is the most effective therapy for reducing the recurrence of heart disease and mortality^1^.

Health-related quality of life (HRQOL) is an individual's subjective perception of physical and mental health^2^. The patient-reported HRQOL is a key indicator of the clinical outcome of treatments, including CABG in patients with CAD^3,4^. Recent studies have shown that the HRQOL as well as symptoms, cardiac functions, and performance of daily activities improved in the majority of patients who underwent CABG^5–7^. However, in some patients, the HRQOL did not return to baseline levels or even worsened after CABG^8,9^. Patients often have post-CABG symptoms such as pain at the incision sites in the chest and leg, respiratory distress, loss of appetite, fatigue, weakness, sleep disturbances, depression, and anxiety^8,10–13^. During the postoperative recovery period, complications, such as wound infections and atrial fibrillation, cause psychological distress^14^ and reduce the patients' HRQOL^6,15^. Moreover, patients experience stress in self-managing their health and implementing lifestyle changes in the post-CABG period to prevent disease progression^16^. These problems adversely affect the patients' ability to return to work as well as adaptation and recovery to normal life^17^.

Furthermore, the HRQOL in the first 12 months after CABG is even more important as this postoperative period involves a transition in the onus of care from the medical staff to the patient and it is important to strengthen the quality of life^6,15^. During this period, changes to the postoperative lifestyle and efforts to reduce risk factors are required^16^. The quality of life in this period can affect the adaptation to life as a CAD patient as well as the long-term quality of life. Therefore, based on the research findings of factors that affect the HRQOL in post-CABG patients, an interventional program to enhance the HRQOL needs to be developed and implemented^10,18^.

Factors that influence the HRQOL of post-CABG patients include: sociodemographics, such as age, sex, medical history, and marital status^3,19^; the presence of emotional symptoms, such as anxiety and depression^8,11,12^; physical symptoms, such as chest pain, dyspnea, fatigue, sleep disturbances, and edema^3^; social support^20–22^; and self-efficacy^23^. Among these factors, post-CABG symptoms significantly affect the patients' HRQOL. Physical symptoms, including postoperative pain and discomfort, are gradually alleviated during the first 4 postoperative weeks^24^. However, emotional symptoms, such as fatigue, sleep disturbances, depression, and anxiety, often persist for longer durations^13^. This persistent symptom experience during recovery increases the risk of recurrence of heart disease as well as hospital readmission and is associated with an increased risk of complications and mortality^25^. Moreover, persistent symptoms that are mediated by factors, such as poor medication adherence, changes in daily life, and adverse effects on social and family relationships, can adversely impact the HRQOL^11,25^.

In contrast to symptom experience, social support and self-efficacy improve the HRQOL^20,21^ and positively influence the recovery process by reducing the depressive symptoms related to poor postoperative recovery^21,22^. Social support prevents and reduces the incidence of stress, helps individuals to adapt psychologically, and positively affects the treatment compliance of CAD patients^20^. Self-efficacy is an individual’s belief in their capability to exercise control over their functioning and on events that affect their lives. Self-efficacy leads to lifestyle change and thus alters health behaviors based on the perceived risk factors for chronic illnesses, such as heart disease^26–28^. Therefore, social support and self-efficacy increase the patient’s confidence in the treatment and psychological resilience^26^, and positively influences the HRQOL^29^.

Nonetheless, despite the importance of HRQOL in the 12 months after CABG, few studies have investigated the HRQOL and the related factors in patients within the 12 months following CABG, which is an important transition period. Therefore, this study aimed to measure the HRQOL levels of patients who underwent CABG within the past 12 months and to determine the factors that are associated with the HRQOL.

## Results

### Participants’ sociodemographics and disease-related characteristics

The overall mean age of participants in the participants was 64.2 ± 9.6 years, and men constituted 81.8% of the study sample. Most of the participants had a spouse (70.9%) and had mid-level economic status (61.8%). Fourty-four participants (40.0%) perceived their health status as good. The most frequent answers to questions on smoking, alcohol consumption, and exercise habits were ex-smoker (61.8%), ex-drinker (44.5%), and irregular exercise (40.0%). Most frequently, the participants had two comorbidities (33.6%, n = 37); moreover, 77.3% of the participants had 3-vessel disease (3VD) and underwent surgery (52.7%) less than 3 months after CABG; this was followed by the proportion of participants who underwent surgery in the 6–12 months (27.3%) and 3–6 months (20.0%) after CABG. (Table 1).

**Table 1:** Sociodemographic and disease-related characteristics of the participants (N = 110).

Variables	Categories	Mean ± SD or n (%)
Age (years, range 42–85)		64.2 ± 9.6
	≤ 60	35 (31.8)
	> 60	75 (68.2)
Gender	Men	90 (81.8)
	Women	20 (18.2)
Education level	≤ Middle school	40 (36.4)
	High school	40 (36.4)
	≥ College	30 (27.2)
Presence of a spouse	Yes	78 (70.9)
	No	32 (29.1)
Perceived economic status	High	4 (3.7)
	Middle	68 (61.8)
	Low	38 (34.5)
Work status	Employed	31 (28.2)
	Unemployed	79 (71.8)
Perceived health status	Good	44 (40.0)
	Average	32 (29.1)
	Poor	34 (30.9)
Smoking	Current smoker	5 (4.6)
	Non-smoker	37 (33.6)
	Ex-smoker	68 (61.8)
Drinking	Current drinker	15 (13.7)
	Non-drinker	46 (41.8)
	Ex-drinker	49 (44.5)
Exercise	No	29 (26.4)
	Irregular	44 (40.0)
	Regular	37 (33.6)
Number of comorbidities	0	21 (19.1)
	1	24 (21.8)
	2	37 (33.6)
	> 3	28 (25.5)
Prior PCI	Yes	32 (29.1)
	No	78 (70.9)
Number of diseased vessels	1	4 (3.6)
	2	21 (19.1)
	3	85 (77.3)
Post-CABG duration (months)	< 3	58 (52.7)
	3–6	22 (20.0)
	6–12	30 (27.3)

*PCI* percutaneous coronary intervention, *CABG* coronary artery bypass graft.

### Participants' symptom experience, self-efficacy, social support, and the HRQOL

The participants' mean of the physical component summary (PCS) and the mental component summary (MCS) scores were 42.56 ± 9.06 and 44.89 ± 11.33 points, respectively. The mean symptom experience and self-efficacy scores were 3.04 ± 3.08 and 2.72 ± 0.74 points, respectively, which—when subdivided into subscales of self-efficacy—comprised 3.09 ± 0.78 and 2.13 ± 1.07 points for symptom control and maintained functioning, respectively. The mean social support score was 2.35 ± 0.64 points, which was subdivided into subscales as follows: 2.36 ± 0.70 for appraisal, 2.42 ± 0.64 for belonging, and 2.29 ± 0.76 for tangible social support (Table 2).

**Table 2:** Descriptive statistics of cardiac symptoms, cardiac self-efficacy, social support, and health-related Quality of Life (N = 110).

Variables	Mean ± SD	Range
**Cardiac symptoms**	3.04 ± 3.08	0–10
**Cardiac self-efficacy**	2.72 ± 0.74	0–4
Control symptoms	3.09 ± 0.78	
Maintain functioning	2.13 ± 1.07	
**Social support**	2.35 ± 0.64	0–3
Appraisal	2.36 ± 0.70	
Belonging	2.42 ± 0.64	
Tangible	2.29 ± 0.76	
**Health-related Quality of Life**		
Physical component summary	42.56 ± 9.06	0–100
Mental component summary	44.89 ± 11.33	0–100

### HRQOL according to the participants' sociodemographic and disease-related characteristics

Based on the sociodemographic and disease-related characteristics of the participants, statistically significant differences in the PCS scores were observed, based on the variables: "presence of a spouse" (t = 1.05, *p* = 0.033), "work status” (t = 14.20, *p* < 0.001), "perceived health status" (F = 8.60, *p* < 0.001), and "post-CABG duration" (F = 5.84, *p* = 0.004). Statistically significant differences in the MCS scores were observed with regard to the variables "perceived health status" (F = 6.18, *p* = 0.003; Table 3).

**Table 3:** Health-related Quality of Life according to characteristics of the patients (N = 110).

Variables	Categories	PCS			MCS		
		Mean ± SD	t or F(p)	Post-hoc	Mean ± SD	t or F(p)	Post-hoc
Age (years)	≤ 60	44.58 ± 7.79	1.58		46.55 ± 11.36	1.03	
	> 60	41.65 ± 9.49	(0.118)		44.15 ± 11.32	(0.307)	
Gender	Men	43.17 ± 8.99	1.52		45.02 ± 11.11	0.26	
	Women	39.79 ± 9.11	(0.131)		44.30 ± 12.59	(0.797)	
Education level	≤ Middle school	40.42 ± 8.18	1.78		45.37 ± 11.63	0.58	
	High school	43.73 ± 9.75	(0.173)		43.40 ± 10.33	(0.559)	
	≥ College	43.86 ± 9.00			46.23 ± 12.34		
Presence of a spouse	Yes	43.73 ± 9.25	1.05		44.12 ± 11.28	0.08	
	No	39.69 ± 8.00	(0.033)		46.76 ± 11.44	(0.270)	
Perceived economic status	High	40.34 ± 12.09	0.94		33.23 ± 12.86	2.61	
	Middle	43.48 ± 9.92	(0.393)		44.65 ± 10.86	(0.078)	
	Low	41.14 ± 6.90			46.54 ± 11.58		
Work status	Employed	47.47 ± 8.13	14.20		44.97 ± 11.53	0.00	
	Unemployed	40.63 ± 8.72	(< 0.001)		44.86 ± 11.34	(0.965)	
Perceived health status	Good ^a^	43.44 ± 8.22	8.60	a,b > c	49.24 ± 9.98	6.18	a > b > c
	Average ^b^	46.33 ± 8.35	(< 0.001)		43.02 ± 10.08	(0.003)	
	Poor ^c^	37.86 ± 8.95			41.03 ± 12.47		
Smoking	Current smoker	43.45 ± 6.29	0.08		51.52 ± 7.49	1.17	
	Non-smoker	42.11 ± 8.76	(0.923)		45.67 ± 11.15	(0.315)	
	Ex-smoker	42.73 ± 9.48			43.98 ± 11.60		
Drinking	Current drinker	40.93 ± 8.95	1.68		45.29 ± 12.78	0.28	
	Non- drinker	45.54 ± 6.74	(0.191)		42.86 ± 8.49	(0.760)	
	Ex-drinker	43.17 ± 9.61			45.14 ± 10.78		
Exercise	No	40.39 ± 8.83	1.65		46.39 ± 11.34	0.44	
	Irregular	44.26 ± 8.76	(0.197)		44.85 ± 12.72	(0.648)	
	Regular	42.23 ± 9.43			43.76 ± 9.63		
Number of comorbidities	0	41.90 ± 10.08	1.69		47.26 ± 10.05	0.92	
	1	42.96 ± 6.51	(0.174)		46.03 ± 11.01	(0.433)	
	2	44.76 ± 9.51			44.87 ± 11.10		
	> 3	39.79 ± 9.18			42.17 ± 12.78		
Prior PCI	Yes	43.46 ± 9.68	0.67		47.61 ± 10.91	1.63	
	No	42.19 ± 8.83	(0.506)		43.77 ± 11.39	(0.107)	
Number of diseased vessels	1	53.30 ± 4.47	3.03		56.89 ± 4.36	2.40	
	2	42.03 ± 8.75	(0.053)		44.07 ± 10.75	(0.096)	
	3	42.18 ± 9.05			44.53 ± 11.47		
Post-CABG duration (months)	< 3^a^	40.36 ± 8.60	5.84	b > c > a	46.30 ± 11.58	0.95	
	3–6^b^	47.75 ± 7.97	(0.004)		43.34 ± 11.14	(0.391)	
	6–12^c^	43.00 ± 9.33			43.30 ± 11.02		

Note: Alphabet (a to c) used as a superscript for categories indicates the alphabet used to describe statistically significance difference. The mean difference is significant at the 0.05 level.

*PCS* physical component summary, *MCS* mental component summary, *PCI* percutaneous coronary intervention, *CABG* coronary artery bypass graft.

### Correlations between the participants' symptom experience, self-efficacy, social support, and the HRQOL

The PCS score was negatively correlated with symptom experience (r =  − 0.476, *p* < 0.001) and was positively associated with self-efficacy (r = 0.461, *p* < 0.001). The MCS score was negatively correlated with symptom experience (r =  − 0.364, *p* < 0.001) and was positively associated with self-efficacy (r = 0.450, *p* < 0.001) and social support (r = 0.285, *p* = 0.003) (Table 4).

**Table 4:** Correlations between cardiac symptoms, cardiac self-efficacy, social support, and health-related Quality of Life (N = 110).

Variables	PCS	MCS
	r(*p*)	r(*p*)
Cardiac symptoms	− 0.476 (< 0.001)	− 0.364 (< 0.001)
Cardiac self-efficacy	0.461 (< 0.001)	0.450 (< 0.001)
Social support	0.139 (0.149)	0.285 (0.003)

*PCS* physical component summary, *MCS* mental component summary.

### Factors that affect the participants' HRQOL

A two-step hierarchical regression analysis was performed to determine the factors that affected the participants' PCS and MCS scores. Before testing the regression model, the underlying assumptions for performing multiple regression analysis were tested, and all assumptions were satisfied. Concerning the PCS, the sociodemographic and disease-related variables that were inputted in Step 1 were: presence of a spouse, perceived health status, work status, and post-CABG duration. These variables explained 27.6% of the variance in the PCS score. Among the input variables, the "presence of a spouse" (B = 4.23, *p* = 0.016), "perceived health status as good" (B = 4.72, *p* = 0.014), " perceived health status as average" (B = 6.55, *p* = 0.002), and the "3–6 months after CABG" (B = 4.96, *p* = 0.025) were identified as factors that were significantly associated with the PCS score. The input of symptom experience and self-efficacy in Step 2 increased the explanatory power by 14.3% up to 41.9% (F = 9.07, *p* < 0.001), and the variables "presence of a spouse" (B = 3.82,* p* = 0.017), "3–6 months after CABG" (B = 5.46, *p* = 0.007), "symptom experience" (B =  − 0.73, *p* = 0.007), and "self-efficacy" (B = 0.25, *p* = 0.005) were identified as factors that affected the PCS score (Table 5).

**Table 5:** Hierarchical regression of physical health-related Quality of Life (N = 110).

Variables	Step I					Step II				
	B	CI	β	t	*p*	B	CI	β	t	*p*
Presence of a spouse (ref: No)	4.23	0.79–7.66	0.21	2.44	0.016	3.82	0.70–6.93	0.19	2.43	0.017
**Perceived health status (ref. Poor)**										
Good	4.72	0.97–8.47	0.26	2.50	0.014	1.75	− 1.86–5.35	0.10	0.96	0.339
Average	6.55	2.55–10.54	0.33	3.25	0.002	3.55	− 0.26–7.37	0.18	1.85	0.067
**Work status (ref: Unemployed)**										
Employed	3.48	− 0.32–7.28	0.17	1.82	0.072	0.74	− 2.87–4.35	0.04	0.41	0.684
**Post-CABG duration (ref. < 3 months)**										
3–6 months	4.96	0.64–9.29	0.22	2.28	0.025	5.46	1.51–9.40	0.24	2.74	0.007
6–12 months	1.22	− 2.54–4.97	0.06	0.64	0.522	1.91	− 1.51–5.33	0.09	1.11	0.270
**Cardiac symptoms**						− 0.73	− 1.27–0.20	− 0.25	− 2.74	0.007
**Cardiac self-efficacy**						0.25	0.08–0.42	0.26	2.87	0.005
R	0.53					0.65				
Adj. R^2^	0.28					0.42				
△ R^2^	0.23					0.37				
F	6.56					9.07				
*p*	< 0.001					< 0.001				

*CABG* coronary artery bypass graft, *CI* confidence interval.

The hierarchical regression analysis that was performed to identify the factors that affected the participants' MCS revealed the sociodemographic and disease-related variables entered in Step 1, perceived health status, which explained 10.0% of the variance in the MCS. A perception of good health status (B = 8.21, *p* = 0.001) was identified as a factor that significantly affected the MCS. The inclusion of symptom experience, social support, and self-efficacy in Step 2 increased the explanatory power by 26.0% to 36.0% (F = 11.53, *p* < 0.001). Among the input variables, social support (B = 0.71, *p* = 0.002) was identified as the strongest influential factor, followed by self-efficacy (B = 0.38, *p* = 0.001) (Table 6).

**Table 6:** Hierarchical regression of mental health-related Quality of life (N = 110).

Variables	Step I					Step II				
	B	CI	β	t	*p*	B	CI	β	t	*p*
**Perceived health status (ref. Poor)**										
Good	8.21	3.31–13.12	0.36	3.32	0.001	5.50	0.84–10.17	0.24	2.34	0.021
Average	2.00	− 3.29–7.29	0.08	0.75	0.455	− 1.48	− 6.43–3.48	− 0.06	−0 .59	0.556
**Cardiac symptoms**						− 0.50	− 1.17–0.18	− 0.14	− 1.45	0.149
**Social support**						0.71	0.28–1.15	0.27	3.25	0.002
**Cardiac self-efficacy**						0.38	2.10–7.75	0.32	3.46	0.001
R	0.32					0.60				
Adj. R^2^	0.10					0.36				
△ R^2^	0.08					0.33				
F	6.18					11.53				
*p*	0.003					< 0.001				

*CI* confidence interval.

## Discussion

This cross-sectional survey-based study was conducted to determine the factors that affect the postoperative HRQOL in patients who are diagnosed with CAD and have undergone CABG. The mean PCS scores were lower than the mean MCS scores (42.56 vs. 44.89), which is similar to the characteristics of PCS vs. MCS scores (42.9 vs. 46.1) that were reported by Kim^30^, who investigated the post-CABG HRQOL of patients and the PCS vs. MCS scores (39.9 vs. 48.9) reported by Verwijmeren et al.^7^ who measured the post-CABG HRQOL at the 12-month timepoint. In the majority of patients, although the post-CABG HRQOL showed improvements compared to the baseline levels^8,19^, the PCS score consistently remained less than the MCS^5,19^. This outscoring of the post-CABG PCS by the post-CABG MCS is attributable to the limitation of everyday physical activity and the decreased treatment compliance rate after CABG^11,25^, which can also negatively affect the MCS^7^. One-fifth of CAD patients have a constantly low PCS score after CABG^9^, which highlights the need for a follow-up study to screen the high-risk group with a persistently low PCS score and follow the changing trends of PCS scores from the immediate post-CABG timepoint up to 12 months.

The hierarchical regression analysis performed to identify the factors that affect HRQOL revealed that the PCS score was significantly affected by the following variables: presence of a spouse, post-CABG period, symptom experience, and self-efficacy. In contrast, the MCS score was affected substantially by self-efficacy and social support. After the CABG, the PCS and MCS scores were most strongly affected by self-efficacy, and this finding is in agreement with the results of previous studies and research which indicated that self-efficacy is a key factor that determined the post-CABG HRQOL in patients with CAD^23^ and heart failure^31^. Recovery and adaptation following CABG are influenced by the patient's physical condition and treatment, as well as by their perception of their own capability to organize and execute the actions that are required to achieve their goals^28,32,33^. This is because self-efficacy helps CAD patients recognize risk factors, thereby enhancing their self-care compliance, such as through positive lifestyle changes^27,28^, confidence in treatment, and psychological resilience^29^. Similarly, regardless of the severity of the disease and depressive symptoms, patients with low self-efficacy had an increased symptom burden and reduced physical functionality and HRQOL^28,34^. It is therefore essential to develop and provide a self-efficacy promotion program that is individualized to CABG patients and includes personal mastery, role modeling, and social support to improve the post-CABG HRQOL.

Symptom experience was identified as a significant factor that affects the PCS score, and this finding is in agreement with the findings of previous studies, which found that symptoms such as depression, anxiety^12^, postoperative adverse effects^9^, angina, and shortness of breath^11,19^ decreased the PCS score of post-CABG patients. The more serious the post-CABG symptom experience, the more likely it is that the uncertainty and supportive care needs will increase^35^. Moreover, perceived symptoms such as angina and shortness of breath lead to movement disorders and limitations in physical activity, which affect the HRQOL^3,5,11,18^. In particular, a negative symptom experience hinders self-care and treatment-seeking behaviors and negatively affects the recovery process and the HRQOL^36^. Therefore, medical staff should periodically measure the degree of symptoms that patients experience after CABG and strive to provide individualized symptom management interventions to improve the HRQOL.

Social support was identified as a major factor that affected the MCS score. The results of this study are aligned with those of a previous study^21^, which reported that emotional or instrumental social support had a positive effect on the MCS scores of post-CABG patients. Depending on the post-CABG recovery process and the patient's conditions, the type and level of a patient's demand for support can vary^21^. However, post-CABG patients experience psychological pain due to multiple postoperative health problems^14^. This should not prevent them from thoroughly implementing lifestyle improvement and medication adherence strategies to prevent disease progression^16^. Social support can improve the MCS score by alleviating emotional stressors, such as depression, and thereby could facilitate the postoperative psychological adaptation and recovery process^20–22^. In addition, social support provides practical help with postoperative maintenance and management of healthy lifestyles, which positively affects the self-care adherence of patients with CAD^20^. Therefore, it is necessary to check the individual patients' support system and provide them with network-based social support, including fellow patients, family members, and medical staff, to enhance their sense of belonging by maintaining emotional bonds and providing practical help through counseling whenever there are difficulties.

Furthermore, the presence of a spouse was identified as an important variable associated with the PCS score, which is consistent with the report that the HRQOL of unmarried patients was significantly lower than that of married patients in a study^3^ which investigated the HRQOL and the influencing factors in 6099 patients who underwent heart surgery (CABG, n = 3679). Having a spouse is a key factor for postoperative HRQOL because a spouse acts as a personal assistant to the patient by assisting in complying with a healthy lifestyle after surgery^10^. Patients often rely on their spouses for help both before and after surgery^37^, and spousal support plays an essential role in reducing risk factors of CAD, thus contributing to psychosocial recovery and mortality reduction^10^. Therefore, it is necessary to include the spouse when setting up long-term therapy and self-management strategies for a post-CABG patient. For example, for an unmarried patient living alone, it is necessary to identify a family member, a friend, or a person who is significant to the patient, who can be the main caregiver—similar to that of the spouse of a married patient—and to include that person in the nursing plan from the preoperative stage to improve the postoperative HRQOL of the individuals.

The post-CABG duration was identified as a factor that affects the PCS score: the HRQOL during the 3–6 month after CABG was significantly higher than that within the 3 months following the surgery, as reported previously^5,6^. This is because the baseline symptoms would have improved in the 3–6 month after CABG and the patient would experience less difficulty in physical activity and thus have a higher PCS score^18^. Within the 3 months after CABG, the recovery process can be affected by postoperative complications (dyspnea, arrhythmia, acute renal failure, stroke, and wound infections) rather than symptom relief and, therefore, the patient's post-CABG HRQOL can be lower than the one at baseline^10,38^. To address this problem, a long-term follow-up of the patient's perceived post-CABG HRQOL needs to be performed to identify the timepoints and periods of decline in the PCS score and, through an analysis of factors that affect the HRQOL, to develop a personalized nursing intervention program that is modulated to the post-CABG period.

This study is significant in that it measured the HRQOL and identified the influencing factors only within the 12 months after CABG. However, the results of this study have a few limitations. First, as the participant enrolment was limited to post-CABG patients treated at the outpatient department of a tertiary-care hospital, caution is warranted in interpreting the results. To enhance the generalizability of the study’s results, it is necessary to perform multicenter, large sample follow-up studies. Second, as this is a cross-sectional study, the participant’s pre-CABG HRQOL could not be ascertained. Therefore, this study has a limitation in that it is not possible to grasp the change in the HRQOL from before to after CABG. A longitudinal study is needed to grasp the change in the HRQOL from before CABG to that at 12 months after CABG. Finally, among the factors that affect the HRQOL are surgical factors such as urgent surgery^9^, complications^15^, and readmission^39^. However, as this study did not investigate these surgical characteristics, caution should be exercised in the interpretation of the study’s results.

## Methods

### Study design and participants

A cross-sectional correlation design was used in this study to determine the effects of symptom experience, self-efficacy, and social support on the HRQOL in post-CABG patients.

The participants were recruited from among CAD outpatients who were regularly followed-up at the Department of Thoracic & Cardiovascular Surgery at a university hospital in Gyeonggi-do, Republic of Korea. The inclusion criteria were: patients between 19 and 75 years of age, with a diagnosis of CAD, having undergone CABG within the preceding 12 months, and able to speak and read Korean. The exclusion criteria were: (1) history of rehospitalization or reoperation due to post-CABG complications; (2) patients who were unable to complete the study tools (e.g., hearing impairment), other associated surgeries (e.g., valvular surgery, CABG with valvular surgery, aortic aneurysm surgery), and (3) patients with major comorbidities, such as stroke, cancer, renal- or liver failure, or communication/psychological limitations that are likely to affect their ability to consent or participate.

The minimum sample size required for regression analysis in this study was 98, when calculated with regard to a median effect size (f^2^) = 0.15, *p* = 0.05, power (1 − β) = 0.80, and with six significant predictors that were suggested in prior research. In the final analysis, 110 respondents were included after excluding two with incomplete responses; we assumed a dropout rate of < 15%.

### Measures

#### Symptom experience

The Cardiac Symptom Survey (CSS) developed by Nieveen et al.^40^ was used to assess the symptom experience in individual patients. The CSS assesses patients' perceptions, evaluations, and responses to 10 symptoms that are commonly experienced by post-CABG patients. First, patients rate their perception of having one of 10 symptoms (angina, shortness of breath, fatigue, depression, trouble sleeping, pain from surgery, swollen legs, palpitation, anxiety, and poor appetite) on a scale of 0 = no and 1 = yes. Then, if a symptom is present, patients rate their symptoms by scoring the frequency and severity of the symptoms on separate scales from 0 to 10, with a rating of 10 indicating the highest frequency or severity. Similarly, the patient can respond to symptoms with regard to interference with their physical activity and enjoyment of life on the same 0 to 10 scale; however, in this study, response to physical activity and enjoyment of life were not included in the calculation of the severity scores. Thus, the evaluation of the symptoms was estimated by calculating a mean score of frequency and severity ratings. The reliability of internal consistency (Cronbach's α) of the CSS score determined at the time of its development was 0.85^40^, whereas, in this study, the Cronbach's α was 0.86.

#### Self-efficacy

Self-efficacy was assessed using the Cardiac Self-Efficacy (CSE) scale, which was developed by Sullivan et al.^34^ to measure the self-efficacy that is perceived by CAD patients. The CSE scale consists of 14 items, in which patients are asked to rate: “how confident are you that you know or can…” on a 5-point Likert scale (0 = not at all, 1 = somewhat confident, 2 = moderately confident, 3 = very confident, 4 = completely confident). The CSE Scale comprises two dimensions (control symptoms and maintain functioning). The control symptom dimension consists of eight items, and the maintain functioning dimension consists of the remaining six items. The internal consistency reliability (Cronbach's α) of the CSE scale was 0.90 for control symptoms and 0.87 for maintenance functions at the time of its development^34^, and 0.86 and 0.85, respectively, in this study.

#### Social support

The Interpersonal Support Evaluation List-12 (ISEL-12), which was developed by Cohen and Hoberman^41^ and translated into Korean by Kim et al.^42^, was used to determine social support. This 12-item scale consists of three subscales: appraisal, belonging, and tangible social support. Each item is rated on a 4-point Likert scale (0 = Definitely false, 1 = Probably false, 2 = Probably true, and 3 = Definitely true). Items 1, 2, 7, 8, 11, and 12 are negative statements that are reverse-scored. The answers to positively worded items are assigned 0–3 points. In contrast, answers to negatively worded items are scored 3–0 points, with the total score ranging between 0 and 36 points, where a higher score indicates higher perceived social support. The internal consistency reliability (Cronbach's α) of this scale at the time of development ranged from 0.88 to 0.90^41^, whereas the reliability in this study was 0.91.

#### Health-related quality of life

The HRQOL was measured using the Korean version of the Short Form-12 Item Version 2 (SF-12v2^Ⓡ^), which was developed by Ware et al.^43^. The SF-12v2, which is a concise version of the Short Form-36 Health Survey Questionnaire, consists of 12 items that pertain to eight domains for QOL measurement (physical functioning, physical role limitations, general health, bodily pain, vitality, social functioning, emotional role limitations, and mental health). The scores for physical and mental HRQOL—that is, the Physical Component Summary (PCS) and Mental Component Summary (MCS) scores—range from 0 to 100, wherein a higher score indicates higher levels of health status and QOL. The Korean version of the SF-12v2 questionnaire was purchased from Quality Metric, Inc. (Lincoln, USA), the copyright holder, after accepting the license agreement (License No. QM053535/OP083363). Furthermore, the SF-12v2-based PCS and MCS scores were calculated using the proprietary program that was provided by Quality Metric, Inc. The internal consistency reliability (Cronbach's α) of the PCS and MCS scores of the SF-12v2 was 0.91 and 0.87^43^ at the time of its development and 0.81 and 0.74 in the present study.

### Data collection

This study meets the ethical standards outlined in the Declaration of Helsinki, and ethics approval was obtained by the institutional review board of Ajou University Hospital (AJIRB-MED-SUR-20). Data were collected from July 01, 2020 to October 31, 2021. Participants were recruited from the outpatients who visited the Department of Thoracic Surgery at the study site based on the recommendations of the cardiologist in charge, per the inclusion criteria. The questionnaire survey was conducted after the researcher explained the purpose of the study to individual potential participants and obtained written informed consent from them. Among the measurement variables, disease-related characteristics were examined through a review of electronic medical records after obtaining consent from each patient. All data were coded and stored in a personal information-storage device. The data files were password-protected to ensure that the research data were inaccessible to anyone other than the researcher.

### Data analysis

The data collected were analyzed using the SPSS WIN 25.0 program. The participants' sociodemographic and disease-related characteristics were analyzed using frequencies, percentages, the mean and standard deviation values. The HRQOL that was associated with the participants' sociodemographic and disease-related characteristics was analyzed using the independent *t*-test and ANOVA, and Scheffe's test was used for post hoc testing. Pearson correlation was used to analyze the correlations among the participants' perceived symptoms, self-efficacy, social support, and the HRQOL. Finally, a hierarchical multiple regression analysis was performed to identify the factors that were associated with the participants’ HRQOL.

## Data Availability

The datasets generated for this study are available on request to the corresponding author.
